# Influenza-Associated Cardiac Manifestations: A Systematic Review of Case Reports and Case Series

**DOI:** 10.3390/jcm15166243

**Published:** 2026-08-12

**Authors:** Sumit Aggarwal, Vikram Singh, Aayushi Bhasin, Sachit Anand, Sanghamitra Pati

**Affiliations:** 1 Indian Council of Medical Research (ICMR) Headquarters, New Delhi 110029, India; 2 All India Institute of Medical Sciences (AIIMS), New Delhi 110029, India

**Keywords:** influenza, myocarditis, cardiac complications, sudden cardiac death, case reports

## Abstract

**Background:** Influenza is primarily a respiratory illness, but it is increasingly linked to acute cardiovascular complications, including myocarditis and sudden cardiac death. Evidence on influenza-associated cardiac involvement remains fragmented and largely derived from case-based reports. **Methods:** This systematic review followed PRISMA guidelines and was registered with PROSPERO (CRD420251249130). PubMed, Web of Science, and Google Scholar were searched for eligible studies. Case reports and case series were included to characterize rare, severe, or atypical cardiac manifestations that are not captured in larger epidemiological studies. Data on demographics, influenza subtype, cardiac manifestations, investigations, management, and outcomes were extracted. **Results:** Thirty-three studies (32 case reports and one case series) comprising 36 cases were included. Influenza A was reported in 26 cases and influenza B was reported in 9 cases. Myocarditis was the most common manifestation (19 cases, 53%), including fulminant myocarditis in 9 cases (25%). Cardiogenic shock occurred in seven cases (19%), malignant arrhythmias in four(11%), myocardial infarction–like presentations in three (8%), and cardiac arrest or sudden cardiac death in six cases (17%). Cardiac involvement occurred across all age groups, frequently in patients without pre-existing coronary artery disease. Overall mortality was 44% (16/36), with fatalities reported in both influenza A and B infections. These findings predominantly reflect severe and published cases and may not represent the full clinical spectrum of influenza-associated cardiac involvement. **Conclusions:** This review highlights that influenza infection has been reported in association with a range of acute cardiac manifestations, particularly myocarditis, including severe and fulminant presentations. However, given the reliance on case reports and case series, the findings are subject to significant publication bias and do not permit the estimation of incidence, risk, or causal inference. These observations should therefore be interpreted as hypothesis-generating.

## 1. Introduction

Influenza is an acute respiratory viral infection that is highly contagious and spreads through droplet infection. Although it is categorized as a seasonal flu that has a low fatality rate, it has marked significant global epidemics in each century: the Spanish flu (1918) [1], extreme intra-sub typic antigenic variation and pseudo pandemic (1947), Asian flu (1957), Hong Kong flu (1968), Abortive, Potentially Pandemic (1976), Swine Influenza Virus Epidemic, 20th century Murky Crystal Ball, and 2009-Swine flu [1]. Currently, influenza accounts for 3–5 million cases of severe illness each year, and the mortality rate falls between 29,000 and 650,000 respiratory deaths annually [2]. While influenza primarily begins with respiratory symptoms, it slowly progresses to extrapulmonary cardiovascular complications that affect the entire body [3].

Influenza virus, a part of Orthomyxoviridae family, includes two main types: influenza A and B viruses that are negatively sensed single-stranded RNA, enveloped viruses circulating humans seasonally [4]. The viral surface glycoproteins hemagglutinin (HA) and neuraminidase (NA) determine virulence, mediate viral entry, release, antigenicity and host adaptation [5]. HA enables viral entry by binding to sialic acid receptors, while NA aids in viral release. These features allow influenza viruses to cause both respiratory and extrapulmonary disease [5]. Influenza A’s high mutation rate, through antigenic drift and shift, allows for immune evasion and drives epidemics [6]. The 2009 pandemic A/H1N1 strain binds α2,6-linked sialic acid receptors in the lower respiratory tract, leading to severe complications like myocarditis [7].

Growing evidence suggests that influenza is associated with a spectrum of ischemic and non-ischemic cardiac events, such as myocardial infarction [8], myocardial injury [9], stroke [10], stress, cardiomyopathy, myocarditis, and pericarditis [11]. The pathophysiology of influenza-associated cardiac complications is likely multifactorial.

It begins with influenza-induced systemic inflammation that elevates pro-inflammatory cytokines (such as interleukin-6 and tumour necrosis factor-alpha), which can destabilise coronary artery plaques and trigger acute myocardial infarction [12]. Second, influenza infection promotes a prothrombotic state by activating platelets, impairing endothelial function, and increasing levels of coagulation factors, thereby increasing the risk of both arterial and venous thrombosis [13]. Third, autopsy studies have demonstrated that influenza virus can directly invade the myocardium, with viral RNA detected in cardiac tissue and resulting in lymphocytic myocarditis [14]. Finally, the hypoxia and heightened metabolic demands associated with influenza-related pneumonia can overwhelm cardiac function, especially in individuals with pre-existing cardiovascular disease [15]. Together, these mechanisms highlight the complex ways in which influenza infection can adversely affect the heart, emphasizing the importance of prevention and early intervention.

The current treatment of influenza includes neuraminidase inhibitors (oseltamivir, zanamivir, peramivir) and the cap-dependent endonuclease inhibitor baloxavir marboxil as key antiviral agents [16,17]. These agents vary in terms of their administration route (oral, inhaled, or intravenous), optimal timing of initiation, and resistance profiles [16,17]. Early antiviral therapy, particularly oseltamivir initiated on the day of hospital admission, improves clinical outcomes in suitable patients, reducing the risk of disease progression, extrapulmonary organ failure, and mortality [18]. Intravenous peramivir has been used successfully in severe influenza-associated myocarditis cases [19]. Supportive care may involve antipyretics, hydration, oxygen supplementation, and, when cardiac complications arise, intensive cardiovascular support including vasopressors, inotropes, and mechanical circulatory support such as extracorporeal membrane oxygenation (ECMO) [20,21,22]. The role of adjunctive corticosteroids in viral myocarditis remains controversial, as current evidence does not demonstrate a mortality benefit despite possible improvements in cardiac function [23].

In this review, the term “cardiac manifestations” refers to clinical cardiac complications occurring during or following influenza infection [5]. The frequency of myocarditis in severely ill patients due to previous seasonal influenza virus infections is reported to be 0.4–13% [24]. Influenza B has historically attracted less research attention than influenza A, partly because of the greater epidemic potential of influenza A [25]. Although both virus types can cause severe cardiac complications, including fulminant myocarditis and sudden cardiac death. Influenza vaccination has been shown to reduce the risk of cardiovascular events associated with influenza infection [26]. Case reports and case series provide valuable insights into rare but clinically significant complications, particularly in conditions such as influenza-associated myocarditis, where large-scale prospective data remain limited. In order to ensure consistency in the case classification, this review adopted standardized diagnostic criteria established by International Cardiology societies. According to the European Society of Cardiology (ESC) 2013 position statement [27] and the American Heart Association (AHA) scientific statement [28], the diagnosis of myocarditis can be categorized as (1) definite (histopathological confirmation), (2) probable (clinical syndrome with supportive biomarkers, ECG, or echocardiographic findings in the absence of obstructive coronary disease), or (3) possible (author-reported diagnosis without sufficient detail).

Although a robust body of research indicates that influenza infection plays a role in the development of cardiovascular disease, the precise mechanisms by which this occurs are not yet fully elucidated. Large-scale epidemiological data on the complete range of cardiac involvement are also limited. Case reports and series, though more likely to focus on severe or unusual cases, offer important perspectives on rare complications that may be missed in broader studies. It is also important to recognise that mild or subclinical myocarditis is likely underdiagnosed and underreported, underscoring the need for greater clinical awareness and research in this area. The objective of this study was to systematically examine influenza-associated cardiac manifestations, with particular emphasis on myocarditis, by synthesizing evidence from published case reports and case series. This review aimed to characterize the spectrum of cardiac manifestations reported in association with influenza, describe clinical presentations and outcomes, and summarize mortality patterns related to influenza-associated cardiac involvement. Additionally, available demographic and clinical characteristics were evaluated to identify factors that may influence the occurrence and severity of these cardiac complications. It is important to note that case-based literature disproportionately represents severe or atypical presentations, while mild or subclinical myocarditis remains underdiagnosed and underreported.

## 2. Methodology

### 2.1. Registration

This systematic review was registered with PROSPERO, the International Prospective Register for Systematic Reviews, and is registered on the online database with the PROSPERO Identifier: CRD420251249130.

### 2.2. Eligibility Criteria

Eligibility criteria were defined using the PECOST framework. The population (P) included human participants of any age or nationality with clinically suspected or laboratory-confirmed influenza infection. The exposure (E) of interest was influenza infection, and the comparator (C) comprised individuals without influenza infection. Outcomes included acute cardiac manifestations, categorized as myocarditis-related, ischemic events (e.g., myocardial infarction), arrhythmic events, and cardiac arrest or sudden cardiac death.

Given the nature of case-based literature, standardized diagnostic criteria were not uniformly applied across studies. For the purpose of this review, the following were used: (Definite myocarditis: Histopathological confirmation (endomyocardial biopsy or autopsy) or cardiac MRI findings consistent with myocarditis, Probable myocarditis: Clinical syndrome (e.g., chest pain, heart failure, arrhythmia) with supportive findings such as elevated cardiac biomarkers, ECG changes, or echocardiographic abnormalities, in the absence of obstructive coronary artery disease, Possible myocarditis: Author-reported diagnosis without sufficient supporting detail).

Regarding study design (S), we included case reports and case series that provided sufficient diagnostic detail for both influenza infection and cardiac involvement. The time component (T) required that cardiac manifestations occurred during the acute phase of influenza infection or within 14 days of influenza symptom onset (or laboratory confirmation if symptom onset was not reported) to support a potential causal association. Only full-text articles published in English were considered. In vitro and animal studies were excluded, as were studies lacking adequate outcome data or reporting cardiac complications following influenza vaccination.

Large observational studies and surveillance reports were excluded because the objective of this review was to provide a detailed characterization of clinical presentation, diagnostic findings, management, and outcomes of individual influenza-associated cardiac cases. Case reports and case series allow for the detailed evaluation of rare, severe, or atypical manifestations that are often insufficiently described in large epidemiological datasets.

### 2.3. Information Sources

This study is conducted in accordance with the PRISMA (Preferred Reporting Items for Systematic Reviews) guidelines. We conducted extensive searches on various databases, including PubMed and Web of Science. Additionally, we conducted a manual search on Google Scholar to identify any missing items and address any gaps in the grey literature or missing information.

### 2.4. Search Strategy

Our search strategy utilised Boolean Operators such as ‘AND’ and ‘OR’, and we included relevant medical subject headings. We designed two distinct search strings for different databases including PubMed and Web of Science.

A search was conducted on 1 September 2025 on Pubmed using the following search string:
(“influenza, human” [Mesh Terms] OR “influenza” OR “flu” OR “respiratory tract infections” [Mesh Terms] OR “respiratory infection”) (“sudden cardiac arrest” OR “sudden cardiac death” OR “cardiac arrest” OR “sudden death” OR “myocardial infarction” OR “coronary event”)

A similar search was conducted on Web of Science on 21 September 2025 using the following search string:
Topic(‘influenza’ OR ‘influenza virus’ OR ‘flu’ OR ‘H1N1’ OR ‘H3N2’ OR ‘respiratory infection*’ OR ‘respiratory tract infection*’) Topic(‘sudden cardiac arrest’ OR ‘sudden cardiac death’ OR ‘cardiac arrest’ OR ‘sudden death’ OR ‘myocardial infarction’ OR ‘coronary event*’ OR ‘cardiac event*’) Topic(‘case report’ OR ‘case series’)

All papers were extracted in RIS file format and uploaded to Rayyan AI, where further screening was performed.

Further, an additional search was conducted on Google Scholar to cover relevant missing literature and grey literature. An article search was performed on 1 October 2025 using search terms such as “influenza and cardiovascular disease”, and the first 250 articles were screened for relevant potential missing or grey literature.

### 2.5. Screening of the Articles

The title and abstract screening were conducted independently by two reviewers (VS and SA) using Rayyan software (Rayyan Systems Inc., Cambridge, MA, USA). Results from both databases, along with articles identified through a manual search on Google Scholar, were screened separately. Each record was categorized as included, excluded, or uncertain. Any discrepancies between reviewers were resolved through discussion, with the involvement of a third reviewer (SP) when necessary. Subsequently, the full-text screening of all potentially eligible studies was performed independently by both reviewers to determine the final inclusion of studies in the review.

### 2.6. Data Extraction & Analysis

Data extraction was carried out independently by two reviewers, each using a standardized data collection form to promote accuracy and reduce bias. The process began with both reviewers working separately on a blinded Excel sheet. After completing their initial extraction, they compared results and discussed any differences to resolve conflicts. The final dataset included detailed information such as study design, title, author, country, gender, age, comorbidities, coronary disease history, flu symptoms, influenza vaccination status, admission symptoms, diagnosis of influenza, vital signs, laboratory findings, ECG results, treatments, length of hospital stay, ICU admission, myocardial symptoms, outcomes, survival or death, and autopsy findings.

A quantitative synthesis (meta-analysis) was not planned or performed due to the descriptive nature of the included studies, which consisted predominantly of case reports and case series with substantial heterogeneity in clinical presentation, diagnostic approaches, influenza subtypes, cardiac manifestations, and reported outcomes. Therefore, the findings were synthesized narratively and summarized descriptively.

### 2.7. Study Risk-of-Bias Assessment

The quality of the included studies was scrutinized by two independent reviewers; the review procedure was blinded between the reviewers to maintain the quality of the appraisal. The Joanna Briggs Institute tool was used to assess the quality of the selected articles. JBI have separate tools for different types of research, including clinical trials, quasi-experimental design, and observational studies (JBI Critical Appraisal Tool (https://jbi.global/critical-appraisal-tools, accessed on 17 December 2025) [29]. Systematic reviews require a critical analysis of the possible risk of bias. The purpose of this appraisal was to assess the methodological quality of a study and to determine the extent to which a study has addressed the possibility of bias in its design, conduct, and analysis. All articles included in this systematic review were subjected to rigorous appraisal by two appraisers/authors.

After independent appraisal, the results were collected and the discrepancies were sorted by discussing with a third reviewer. The questions in the checklist have four responses; Yes, No, Not Applicable (N/A), and Unclear. The answer is provided by the checkbox as per the most appropriate answer: yes if the criteria are accepted by the reviewer, no if the clarification is not adequate, N/A if the criteria are not applicable, as per the research type, and unclear if the detail is not provided in the article.

Methodological quality was assessed using the Joanna Briggs Institute (JBI) Critical Appraisal Checklist for Case Reports, which comprises eight domains (D1–D8). Each domain corresponds to specific methodological questions, which are as follows:

D1: Were patient’s demographic characteristics clearly described?

D2: Was the patient’s history clearly described and presented as a timeline?

D3: Was the current clinical condition of the patient on presentation clearly described?

D4: Were diagnostic tests or assessment methods and the results clearly described?

D5: Was the intervention(s) or treatment procedure(s) clearly described?

D6: Was the post-intervention clinical condition clearly described?

D7: Were adverse events (harms) or unanticipated events identified and described?

D8: Does the case report provide takeaway lessons?

## 3. Results

**Figure 1:**
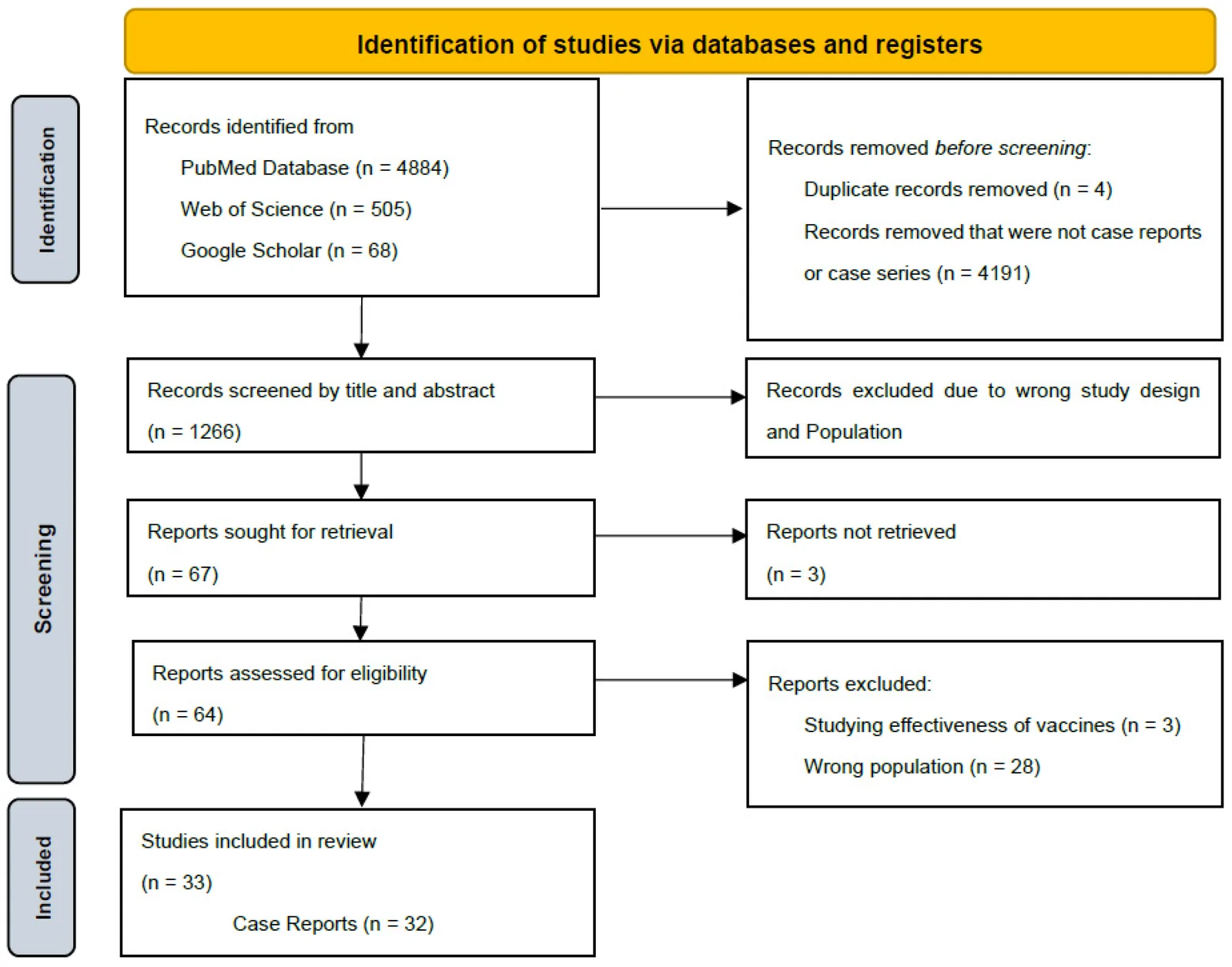
The PRISMA workflow diagram.

In this study the cardiac manifestations were categorized into myocarditis-related, ischemic, and arrhythmic presentations. The relation between influenza infection and cardiac events was examined across a total of 33 studies, comprising 32 case reports and one case series reporting four individual cases. A systematic search of PubMed, Web of Science, and Google Scholar identified 5457 records (Figure 1). After the removal of 4191 records deemed irrelevant due to the study design and four duplicate records, 1262 articles were screened for title and abstract screening using the Rayyan platform. Of these, 1195 articles were excluded based on the predefined eligibility criteria. Further, 67 reports were sought for retrieval; of these, 3 reports were not retrieved as they were conference abstracts without full text or retracted articles. Sixty-four full-text articles were subsequently assessed for eligibility, following which 31 records were excluded because they focused on vaccine effectiveness (*n* = 3) or involved an ineligible study population (*n* = 28). Ultimately, 33 studies met the inclusion criteria and were included in the final synthesis, comprising 32 case reports and 1 case series. The PRISMA 2020 checklist is provided in Supplementary Table S3 [30].

### 3.1. Study Characteristics

This systematic review included 33 studies involving 32 case reports and one case series, comprising a total of 36 individual cases. The majority of the cases were adults aged 18–59 years (28 cases; 78%), comprising 13 males (46%) and 15 females (54%). Pediatric cases were less common; the 0–9 years age category accounted for 4 cases (11%) of cases, with a predominance of females (75%). Only two cases were observed among the adolescents aged 10–17 years, both males. Older adults were minimally represented, with only two cases (6%) documented in individuals over 60 years of age, both of whom were male (Table 1).

In the included studies two major influenza virus types were identified across the reported cases: Influenza A (*n* = 26) and Influenza B (*n* = 9). In one study [31], Influenza B (B/Victoria/2/87-line) RNA was detected in postmortem cardiac tissue using RT-PCR, whereas Influenza A (H2N2) RNA was identified exclusively in tracheal tissue via type-specific PCR. Subtype information was available for 18 of the 36 cases. Among those infected with Influenza A, the majority (14 cases; 82%) were attributed to the A/H1N1pdm2009 strain, while three cases (18%) involved the A/H3N2 subtype.

**Figure 2:**
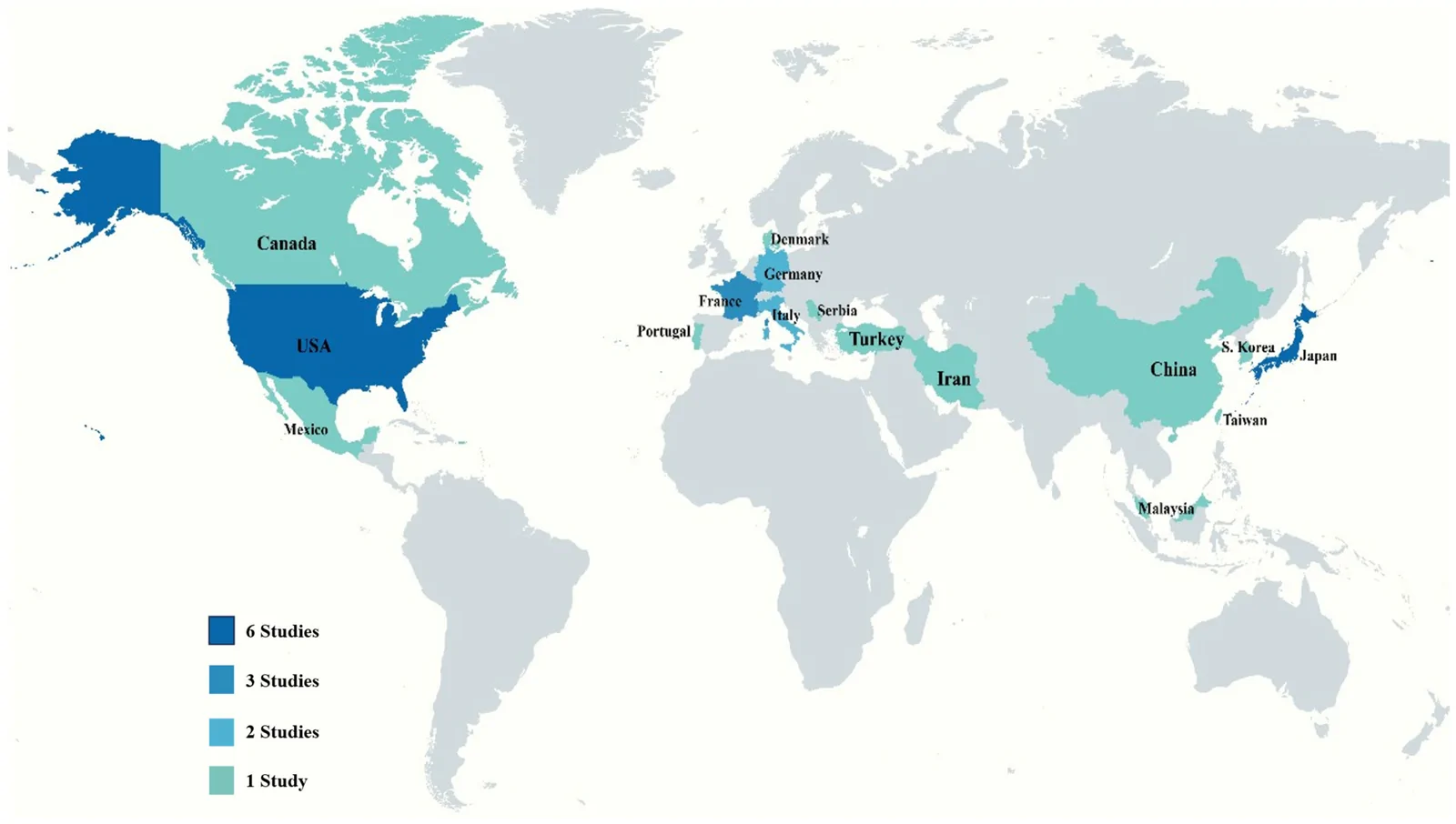
Geographical distribution of included studies.

The studies included in this review were published and originated from a diverse set of countries, reflecting the global occurrence of influenza-associated cardiac events. The most studies included in this review were reported from the United States (7 cases, 19%), followed by Japan (5 cases, 15%), and France (3 cases, 8%). Other studies were from Italy (2), Germany (2), and single reports each from Iran, Turkey, Taiwan, Denmark, China, Canada, Switzerland, South Korea, Brazil, Malaysia, Portugal, Serbia, Mexico, and Puerto Rico (Figure 2). In addition to individual case reports, one case series was identified, each describing multiple patients with influenza-related cardiac events. Overall, most reports originated from high-income countries, suggesting diagnostic capacity or better reporting systems.

Information on prior influenza vaccination status was inconsistently reported across the included studies. Among the 36 cases, 12 patients (33%) were explicitly documented as not having received influenza vaccination, while in 24 cases (67%), vaccination status was not reported. None of the reviewed reports documented a history of prior influenza immunization.

This finding highlights a notable gap in vaccination documentation within case reports and underscores the need for more consistent reporting, particularly given the potential protective role of influenza vaccination against severe cardiac complications such as myocarditis.

### 3.2. Risk-of-Bias Assessment

Among the 32 included case reports, 27 studies were classified as having a low risk of bias, while 5 case reports were categorized as having a moderate risk of bias due to one or more unclear/unmet appraisal criteria [32,33,34,35,36,37]. No case report was classified as having a high risk of bias. The single included case series [38] fulfilled almost all JBI appraisal criteria and was therefore judged to have a low risk of bias overall.

While most studies adequately reported clinical presentation and diagnostic confirmation, deficiencies were observed in intervention- and outcome-related domains. Specifically, Study (Boulagnon et al. 2012) [36] was rated Unclear in D3 and “No” in D5–D6 and [33] received “No” ratings for D5–D6 due to absent treatment and post-intervention outcome details. One study [38] was appraised as a case series using the JBI Critical Appraisal tool for case series. The study(Erden et al. 2011) [32] did not report the follow-up duration or adverse eve nts (D7–D8), while Mazzitelli et al. 2019 [34] and Kovačević et al. 2019 [39] had Unclear ratings for D5 and D6, respectively. All relevant methodological domains were adequately reported in the study, with the exception of D10, which was not available. The case series demonstrated acceptable methodological quality, consistent with its descriptive design.

**Table 1:** Study characteristics.

S. No.	Authors	Country	Gender	Age	History of Coronary Disease	Influenza Vaccin Ation	Influenza Type	Subtype	Hospital Stay Duration	ICU Admission	Myocardial Symptoms Presented
1	Iwanaga et al. 2014 [24]	Japan	Male	52	No	No	Inf A	(H1N1) pdm2009	12 days (Death)	Yes	Acute myocardial Infarction
2	Shakerian and Mandegar 2024 [40]	Iran	Female	34	No	No	Inf A	Not subtyped	1 day (Death)	Yes	Fulminant myocarditis resulting in cardiogenic shock
3	Erden et al. 2011 [32]	Turkey	Female	34	No	NR	Inf A	(H1N1) pdm2009	14 days	NR	Acute Myocarditis
4	Ishiguro et al. 2019 [41]	Japan	Male	58	Inferior LV wall myocardial Infarction and hypertension	No	Inf A	Not subtyped	18 days	NR	Myocardial Infarction
5	Okada et al. 2017 [42]	Japan	Male	55	NR	NR	Inf A	(H1N1) pdm2009	11 days (Death)	Yes	Cardiac arrest
6	Ono et al. 2023 [43]	Japan	Male	38	No coronary risk factors or cardiac history	NR	Inf A	Not subtyped	26 days	Yes	Cardiac arrest
7	Callon et al. 2021 [33]	France	Female	5	No	NR	Inf A	Not subtyped	One day (Death)	No	Cardiac arrest
8	Boulagnon et al. 2012 [35]	France	Male	19	No	NR	Inf A	(H1N1) pdm2009	NA (Death)	NA	Sudden Cardiac death
9	Marchetti et al. 2019 [34]	Italy	Male	44	NR	No	Inf B	Not subtyped	17 days	Yes	Myocarditis with cardiogenic shock
10	Chang et al. 2016 [44]	Taiwan	Male	43	No	NR	Inf B	Not subtyped	35 days	Yes	Acute myocarditis
11	Siskin et al. 2017 [45]	USA	Female	22	No	NR	Inf B	Not subtyped	8 days	No	Cardiogenic shock mediated by Inf B induced myopericarditis
12	Roto et al. 2018 [46]	USA	Female	57	NR	NR	Inf B	Not subtyped	one day (Death)	Yes	Myocarditis
13	Dickey, Schweir, and Hysell 2018 [47]	USA	Female	34	NR	NR	Inf B	Not subtyped	one day (Death)	Yes	Myocarditis led to pulseless ventricular tachycardia and death
14	Hollowed and Nsair 2019 [48]	USA	Male	23	No	No	Inf A	(H3N2)	18 days	No	Severe myocardial Edema
15	Ebdrup et al. 2018 [49]	Denmark	Male	52	A history of localized oedema and increased hematocrit	NR	Inf A	Not subtyped	41 days	Yes	Cardiac arrest
16	Wattanachayakul et al. 2024 [50]	USA	Male	80	No	NR	Inf A	Not subtyped	5 days (Death)	Yes	Acute myocardial Infarction, acute myocarditis, and pulmonary embolism
17	Taremi et al. 2013 [51]	USA	Female	52	No	NR	Inf B	Not subtyped	15 days	Yes	Fulminant myocarditis with cardiogenic shock
18	Muneuchi et al. 2009 [52]	Japan	Male	15	No	NR	Inf B	Not subtyped	7 days	No	Focal myocarditis
19	Tian et al. 2024 [53]	China	Male	7	No	NR	Inf B	Not subtyped	10 days (Death)	Yes	Cardiac arrest
20	Frank et al. 2010 [31]	Germany	Female	5	No	No	Inf B and Inf A	B Victoria/H2N2	One day (Death)	Yes	Fulminant aggravation
21	Murnaghan et al. 2022 [54]	Canada	Male	53	No	No	Inf B	Not subtyped	12 days	Yes	Myocarditis and cardiogenic shock
22	Baik et al. 2015 [19]	South Korea	Female	45	No	No	Inf A	(H3N2)	6 days	Yes	Fulminant myocarditis
23	Mazzitelli et al. 2019 [35]	Italy	Female	23	NR	NR	Inf A	(H1N1) pdm2009	34 days	Yes	Severe Myocarditis
24	Lobo et al. 2014 [55]	Brazil	Female	4	No	NR	Inf A	(H1N1) pdm2009	Two days (Death)	Yes	Fulminant acute myocarditis presents with sudden onset of cardiac symptoms
25	Chang et al., 2020 [56]	Malaysia	Female	32	No	NR	Inf A	Not subtyped	NR	Yes	A diagnosis of myocarditis due to Inf A was made
26	Montcriol et al. 2008 [57]	France	Female	26	No	NR	Inf A	Not subtyped	26 days	Yes	Fulminant myocarditis
27	Cabral et al. 2012 [58]	Portugal	Male	10	No	No	Inf A	(H1N1) pdm2009	NR (Death)	Yes	Sudden asystolic cardiac arrest occurred, fulminant viral myocarditis.
28a	Wiegand et al. 2011 [38]	Switzerland	Female	55	a self-limited episode of palpitations one month earlier.	NR	Inf A	(H1N1) pdm2009	10 days	Yes	Ventricular tachycardia and cardiogenic shock
28b	Wiegand et al. 2011 [38]	Switzerland	Male	59	multiple myeloma, retinitis pigmentosa, arterial hypertension, atrial fibrillation	NR	Inf A	(H1N1) pdm2009	One day (Death)	Yes	Refractory cardiogenic shock
28c	Wiegand et al. 2011 [38]	Switzerland	Female	43	NR	NR	Inf A	(H1N1) pdm2009	14 days	Yes	Ventricular fibrillation
28d	Wiegand et al. 2011 [38]	Switzerland	Female	29	NR	NR	Inf A	(H1N1) pdm2009	28 days	Yes	Acute left heart failure
29	Kovačević et al. 2019 [39]	Serbia	Male	44	No	NR	Inf A	(H3N2)	One day (Death)	Yes; cardiology intensive care unit	Fulminant myocarditis
30	Arbit et al., 2017 [37]	Mexico	Male	21	No	No	Inf A	(H1N1) pdm2009	NR	NR	Myocardial Infarction
31	Gdynia et al. 2011 [59]	Germany	Female	18	No	No	Inf A	(H1N1) pdm2009	One day (Death)	ER admission; no specific ICU admission mentioned	Fatal interstitial myocarditis
32	Rivera-Guzmán et al., 2016 [60]	Puerto Rico	Male	85	Yes	NR	Inf A	Not subtyped	16 days	Yes	Transient atrioventricular (AV) block with
33	Mel A Ona et al. 2012 [61]	USA	Female	38	NR	No	Inf A	(H1N1) pdm2009	NR (Death)	Yes; mechanical ventilation	Fatal Fulminant Myocarditis

**Figure 3:**
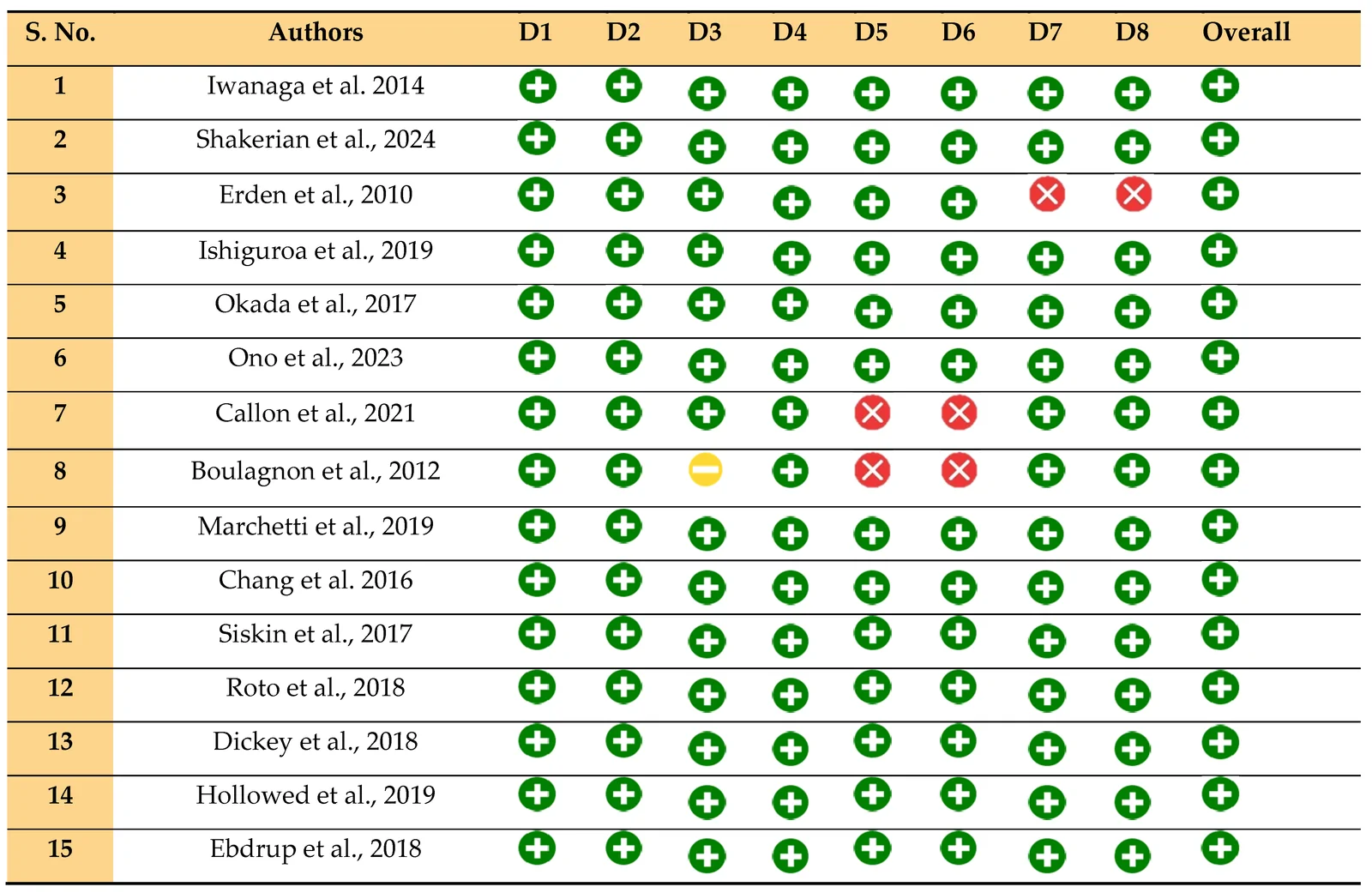

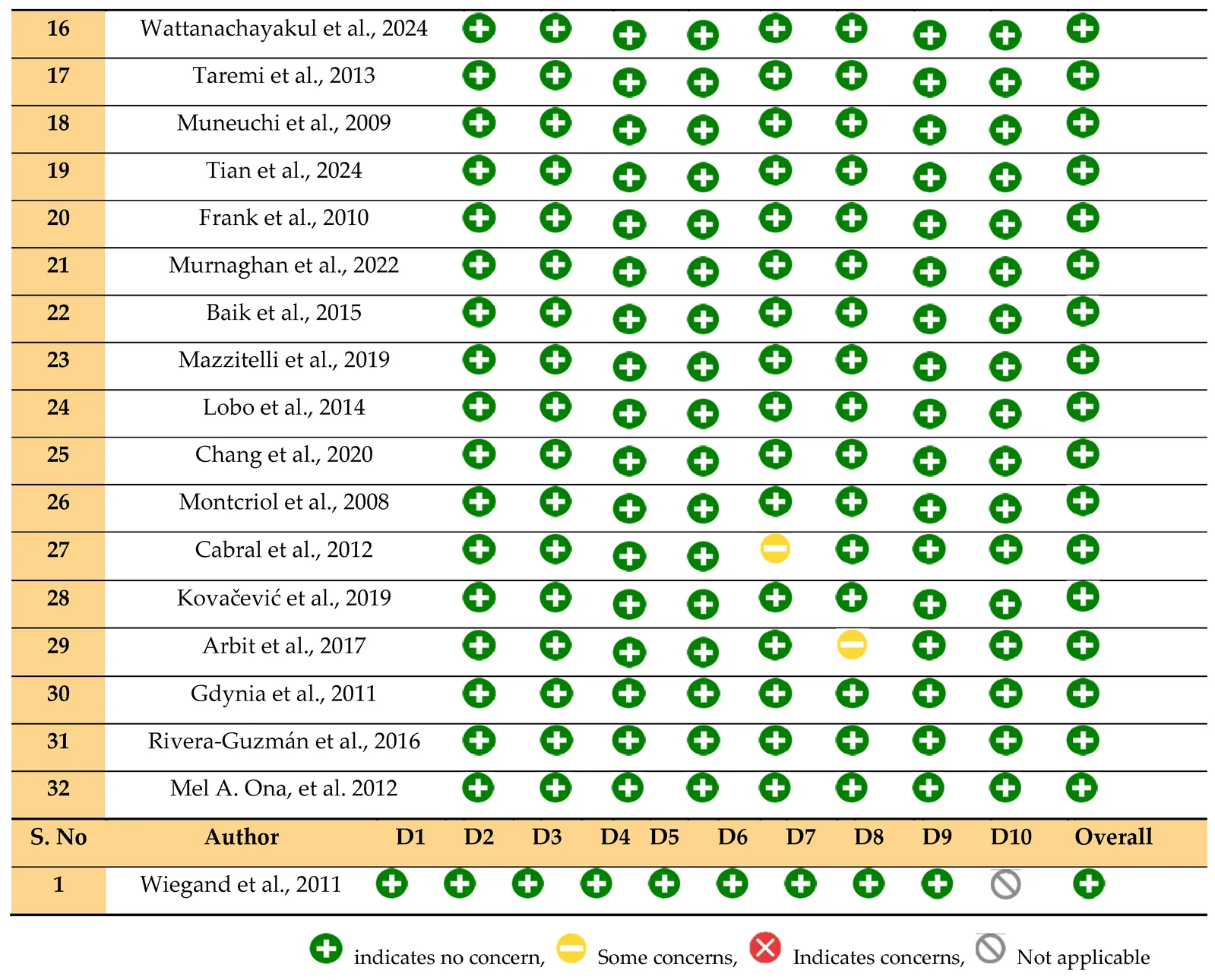
Risk-of-bias assessment. Studies assessed include Iwanaga et al. (2014) [24], Shakerian et al. (2024) [40], Erden et al. (2011) [32], Ishiguro et al. (2019) [41], Okada et al. (2017) [42], Ono et al. (2023) [43], Callon et al. (2021) [33], Boulagnon et al. (2012) [36], Marchetti et al. (2019) [34], Chang et al. (2016) [44], Siskin et al. (2017) [45], Roto et al. (2018) [46], Dickey et al. (2018) [47], Hollowed et al. (2019) [48], Ebdrup et al. (2018) [49], Wattanachayakul et al. (2024) [50], Taremi et al. (2013) [51], Muneuchi et al. (2009) [52], Tian et al. (2024) [53], Frank et al. (2010) [31], Murnaghan et al. (2022) [54], Baik et al. (2015) [19], Mazzitelli et al. (2019) [35], Lobo et al. (2014) [55], Chang et al. (2020) [56], Montcriol et al. (2008) [57], Cabral et al. (2012) [58], Kovačević et al. (2019) [39], Arbit et al. (2011) [37], Gdynia et al. (2011) [59], Rivera-Guzmán et al. (2016) [60], and Ona et al. (2012) [61].

Overall, the evidence demonstrates a moderate risk of bias, primarily driven by incomplete reporting rather than methodological inadequacy, consistent with case-based literature (Figure 3).

### 3.3. Symptoms Reported in Included Studies

#### 3.3.1. Flu-like Symptoms

Across the 33 included case reports, influenza-associated myocarditis most often began with a typical influenza-like illness, with fever being the predominant symptom. High or moderate fever was reported in the majority of cases [32,34,40,51,54,59], although a few patients were afebrile [39,50] (Table S1).

#### 3.3.2. Respiratory Symptoms

Respiratory symptoms were frequent, most commonly cough, which ranged from mild [46,59] to severe and productive [19]. Several patients had URTI features like sore throat, rhinorrhea, or pharyngitis [31,32,61]. Some presented with non-specific influenza-like malaise without prominent respiratory involvement [24,33].

Myalgia, malaise, or fatigue were also common early symptoms, documented across multiple reports [31,32,40,51,54,59].

More clinically concerning features included dyspnea, reported in several cases [35,40,41,42,62], and chest pain, which was noted particularly in cases progressing to fulminant myocarditis or myocardial injury [36,54,62].

#### 3.3.3. Gastrointestinal Symptoms

A subset of patients presented with gastrointestinal manifestations, including nausea, vomiting, diarrhea, or abdominal pain [32,34,47,48,49,55], occasionally constituting the predominant symptom cluster [48].

#### 3.3.4. Neurological Symptoms

Neurological symptoms were uncommon but included altered consciousness [35] and headache [34,38]. Unique features such as hemoptysis [19], excessive sweating [39], and capillary leak syndrome [49] were reported in isolated cases.

#### 3.3.5. Other Clinical Symptoms at Admission

Across the 33 included reports, patients presented with a wide spectrum of symptoms at admission, reflecting both typical influenza-like illness (ILI) and early cardiac compromise. Respiratory symptoms were among the most frequently documented, with dyspnoea or respiratory distress reported in several cases [24,35,39,40,41,50,54,59]. Many patients presented after days of persistent cough, fever, or malaise characteristic of influenza, consistent with the classical prodromal phase described in prior influenza myocarditis literature [41,59].

Similarly, profound hypotension, tachycardia, and signs of shock were noted at presentation in various studies [34,35,40,51]. In several instances, patients arrived in extremis already experiencing cardiac arrest [43,48,49,56], severe dehydration [34,55], or altered mental status [46]. These forms of circulatory collapse are well-recognized in the fulminant myocarditis phenotype following influenza, particularly H1N1 and influenza B subtypes.

Gastrointestinal manifestations including abdominal pain, nausea, vomiting, or diarrhea were relatively common [39,41,43,45,48,49,50], reflecting the systemic inflammatory response often described in pediatric and adult influenza myocarditis cohorts. Some cases presented with isolated GI symptoms before cardiac decompensation, underscoring that non-respiratory influenza manifestations can mask evolving myocardial involvement [47].

Chest pain, either ischemic-like or atypical, was reported in several cases [32,37,39,45,50]. Neurological symptoms such as syncope, lightheadedness, or seizures [19,54,61] were reported in a few cases and likely reflected transient arrhythmias or severe hemodynamic compromise. Several pediatric cases presented with non-specific signs including pallor and dry mucous membranes [31,33], consistent with patterns reported in influenza-associated myocarditis in children.

Overall, these cases demonstrate that while influenza myocarditis often begins with non-specific viral symptoms, progression to dyspnea, chest pain, or hemodynamic instability may occur rapidly. Notably, several severe cases initially presented with only mild or flu-like symptoms, emphasizing the need for vigilance during influenza seasons.

Altogether, the admission presentations reveal that while many patients initially showed classic ILI symptoms, a substantial proportion exhibited early features of cardiac dysfunction, shock, or arrhythmia at the time of evaluation. These findings mirror established clinical descriptions of influenza-associated myocarditis, where rapid clinical deterioration can occur even in previously healthy individuals (Table S3).

### 3.4. Vital Signs at Admission

Vital-sign reporting across the 36 included cases was inconsistent. Among cases reporting heart rate (*n* = 22), tachycardia was common (mean HR 110.6 bpm; median 120 bpm), with 15/22 (68.2%) meeting the criterion for tachycardia (>100 bpm) (Table S2). Systolic blood pressure values exhibited marked variability (*n* = 26; mean = 100.08 mmHg, range 30–146 mmHg), and 8/26 (30.8%) of patients were hypotensive on presentation (SBP < 90 mmHg), including several with profoundly low arterial pressures. Respiratory compromise was notable: the mean respiratory rate was 27.0 breaths/min (*n* = 14), and 9/14 (64.3%) were tachypneic (>20/min). Fever (≥38.0 °C) was documented in 4/16 (25.0%), whereas peripheral oxygen saturation, when recorded (*n* = 9), had a mean of 96.4%; 3/9 (33.3%) of measured saturations were <95%. In summary, tachycardia and tachypnoea were prominent features among reported influenza-associated cardiac cases, while hypotension and hypoxemia were present in clinically meaningful subsets. Interpretation is limited by the incomplete and heterogenous reporting of vital signs.

### 3.5. History of Coronary Disease

A total of 33 case reports, including subcases, describing influenza-related myocarditis or cardiac involvement were analyzed. Survival outcomes were heterogeneous, with both fatal and non-fatal cases documented across diverse clinical presentations. A review of the patients’ cardiovascular histories demonstrated that the majority had no known coronary artery disease or established cardiac risk factors. Only a small number of cases reported pre-existing conditions such as hypertension, atrial fibrillation, prior myocardial infarction, or systemic illnesses that could indirectly affect cardiac function (Ishiguroa et al., 2019; Wiegand et al., 2011) [38,41].

Importantly, fatal outcomes were observed among patients irrespective of coronary history, including several individuals with previously normal coronary arteries confirmed at autopsy. Cases reporting acute thrombotic occlusion typically described new, infection-associated events, rather than the progression of chronic coronary disease. Similarly, several survivors also had no identifiable cardiac risk factors, suggesting that outcomes were influenced more by the severity of acute viral cardiac injury than by baseline coronary status.

Overall, the evidence from these case reports indicates that pre-existing coronary disease was uncommon and did not demonstrate a consistent association with mortality. The cardiac complications described appear predominantly driven by acute myocarditic and inflammatory mechanisms related to influenza infection, rather than underlying chronic coronary pathology.

### 3.6. Cardiac Manifestations Reported

Across the 33 included cases, influenza infection was associated with a broad spectrum of acute cardiac manifestations, with myocarditis being the predominant presentation. Cardiac involvement occurred in both influenza A and B infections, across all age groups (4–85 years), and in patients with and without documented pre-existing coronary artery disease [Refer Table 2].

Myocarditis was reported in 19 cases (53%), including fulminant myocarditis in 9 (25%) cases. Fulminant presentations occurred in both adults and children, most frequently in individuals without known coronary disease, and were commonly associated with rapid clinical deterioration and high mortality. Several fatal cases occurred within 1–2 days of hospital admission. Myocarditis was reported in 10 of 26 cases (38%) involving influenza A infection, including the A/H1N1pdm2009 and H3N2 subtypes, and in 8 of 9 cases (89%) involving influenza B infection. Fatal outcomes were observed with both virus types, occurring in 5 cases (50%) among patients with influenza A associated myocarditis and in 2 cases (25%) among those with influenza B associated myocarditis.

Cardiogenic shock was documented in 7 cases (19%), typically in association with myocarditis, and occurred in both influenza A and B [40,44,50,54,59]. Acute heart failure [37], including left ventricular dysfunction [37], was also reported, occasionally as the initial cardiac manifestation. Survivors often required prolonged hospitalization, whereas fatal cases were characterized by very short hospital stays.

Clinically significant arrhythmias occurred in four cases (11%), including ventricular tachycardia [37,49] ventricular fibrillation [37], and advanced conduction abnormalities [37]. These events were most often observed in fulminant cases and were frequently associated with cardiac arrest or death. A case of transient atrioventricular block was reported in an elderly patient with underlying cardiac disease [45].

Cardiac arrest and sudden cardiac death were reported in eight cases (22%) in both pediatric and adult patients, frequently in the absence of prior coronary disease [32,33,35,63]. Similarly, myocardial infarction was reported in three cases (8%) [24,36,56], occurring in patients both with and without known coronary disease, sometimes with angiographic coronary abnormalities, highlighting diagnostic overlap with acute coronary syndromes.

Overall, influenza-associated cardiac involvement encompassed myocarditis (including fulminant forms), cardiogenic shock, acute heart failure, malignant arrhythmias, myocardial infarction–like presentations, cardiac arrest, and sudden cardiac death. These manifestations were observed irrespective of prior coronary disease and across influenza virus types, underscoring the breadth and severity of cardiac complications reported at the case level.

### 3.7. ECG Findings

Electrocardiographic (ECG) abnormalities were reported across the majority of influenza-associated cardiac events included in this systematic review. ST-segment elevation (STE) was the most frequently documented abnormality, observed in 14 cases (38.9%), often presenting with localized or diffuse involvement of inferior, anterior, lateral, or mixed myocardial territories. These patterns were consistently described across multiple reports and were frequently interpreted in the context of acute myocarditis or influenza-triggered myocardial injury [24,32,34,36,37,39,40,41,43,45,47,50,53,59,61]. Reciprocal ST-segment depression was noted in three cases (8.3%), typically as an accompanying finding in patients with inferior STE, reflecting secondary ischemic changes [32,40,52].

Sinus tachycardia was the second most common ECG pattern, present in nine cases (25%), with or without associated ST–T changes. This nonspecific but clinically relevant finding has frequently been reported in influenza myocarditis, particularly in younger patients or those presenting with systemic inflammatory responses [39,40,46,50,54,55,61]. (Table 3).

Life-threatening ventricular arrhythmias were relatively uncommon but clinically significant. Wide-complex tachycardia or ventricular tachyarrhythmias were identified in two cases (5.6%), typically preceding hemodynamic collapse or sudden cardiac arrest [24,61]. A rare Brugada-pattern ECG (type 1 morphology) was documented in one case, raising the possibility that febrile states induced by influenza may unmask latent channelopathies [43]. Right bundle branch block (RBBB) was reported in two patients, sometimes accompanied by ST-segment elevation in V1–V2, mimicking Brugada phenotypes [34,43].

Other isolated ECG abnormalities included low QRS voltage (1 case, 19), frequent premature ventricular contractions (PVCs) (1 case, 41), and electromechanical dissociation (EMD) (1 case, 43), the latter representing an agonal rhythm associated with pulseless collapse in fulminant myocarditis.

Importantly, three cases (8.3%) demonstrated a normal ECG at initial presentation, despite the subsequent confirmation of myocarditis or severe cardiac involvement, highlighting the potential for influenza-associated myocardial injury to manifest with minimal or absent electrical abnormalities in the early stages [35].

In addition to this, ECG findings were not reported or not sufficiently described in six cases (17%), limiting the interpretation of electrophysiological manifestations in a substantial proportion of published reports [33,36,38,42,49,62]. This underscores a recurrent gap in case report documentation, despite ECG being a readily accessible diagnostic tool in suspected myocarditis.

### 3.8. Number of Mortalities Among Included Cases

**Figure 4:**
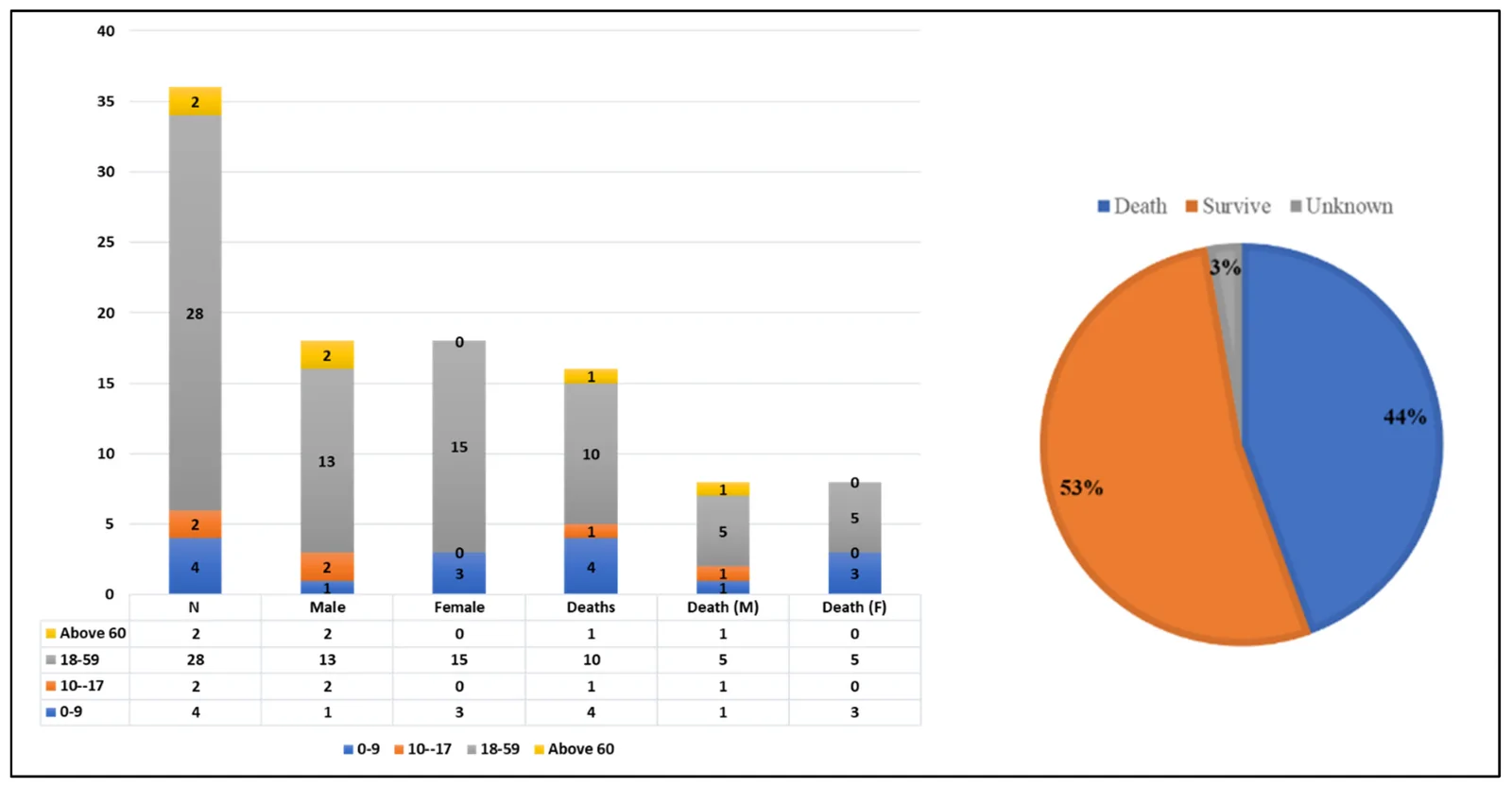
Age-group distribution of total cases and deaths by sex.

Among the 36 included cases, 16 patients died, 19 patients survived, and 1 study (Ishiguroa et al., 2019) [41] did not report the patient’s outcome. Mortality was observed across all age groups, including young children (*n* = 4 deaths below 10 years), adolescents (*n* = 1), adults aged 18–59 years (*n* = 10), and older adults ≥ 60 years (*n* = 1). With respect to sex distribution, fatal cases included 8 males and 8 females, indicating no clear sex-predominant pattern in mortality. Ages among the deceased ranged from 4 to 80 years, with deaths occurring in both otherwise healthy individuals and those with underlying structural abnormalities identified only at autopsy. Most deaths were attributed to Influenza A infection (*n* = 12), whereas Influenza B was implicated in three deaths; one pediatric case involved co-infection with both influenza A and B. Survival was reported more frequently in cases associated with Influenza B, while Influenza A accounted for a larger proportion of severe and fulminant presentations (Figure 4).

### 3.9. Autopsy Findings Among Fatal Cases

Autopsy information was available for most of the fatal cases (14, 87.5%), revealing heterogeneous pathological changes involving the myocardium, lungs, or underlying cardiac structures. A substantial proportion of deaths were attributed to acute myocarditis [31,46,50,55,61], which was the most consistent finding across both pediatric and adult cases. These myocarditis-related fatalities demonstrated diffuse or multifocal lymphocytic infiltration of the myocardium accompanied by myocyte necrosis and interstitial edema [43]. The inflammatory infiltrates frequently consisted of lymphocytes and macrophages, with occasional eosinophils or multinucleated cells [33,34,36,39,40,61]. Such features were reported in children as young as 4, 5, and 10 years, as well as in adults aged 18–59 years. In several reports, including those by [40,46,61], the autopsy findings unequivocally supported influenza-associated myocarditis as the primary cause of death.

Fatal outcomes were also reported in previously healthy young adults [24,36] middle-aged adults [40,47], older adults [50], and multiple children under the age of 10 [31,33,53,55]. Survival, however, was also common even in individuals presenting with fulminant myocarditis physiology [19,32,38,54].

Autopsy findings demonstrated a wide pathological spectrum. Several fatal cases revealed classic lymphocytic myocarditis, characterized by diffuse or patchy lymphocytic infiltration, myocyte necrosis, and interstitial edema [39,40,46,61]. This aligns with established pathology of viral myocarditis described in earlier influenza epidemics. Some cases demonstrated myocardial necrosis or infarction-like changes [24] despite the absence of significant coronary atherosclerosis, suggesting an inflammatory or microvascular injury mechanism rather than obstructive coronary disease consistent with prior mechanistic hypotheses for influenza-induced myocardial injury.

In pediatric deaths, autopsies frequently showed severe bronchiolitis, diffuse alveolar damage, pulmonary hemorrhage, or systemic inflammatory involvement [31,33,55]. These findings parallel historical descriptions of influenza-mediated multi-organ injury in children and highlight that myocarditis may coexist with overwhelming respiratory pathology.

Conversely, several fatal cases displayed minimal or absent myocardial inflammation, instead showing isolated respiratory findings [50], reflecting that influenza can precipitate cardiac arrest through mechanisms beyond overt myocarditis such as severe hypoxia, arrhythmogenic effects, or cytokine-driven collapse.

Notably, only a minority of patients had pre-existing coronary or cardiovascular disease, and several fatal cases occurred in individuals with no coronary risk factors [32,34,39,45]. Mild coronary sclerosis, hypertension, or unrelated comorbidities were observed sporadically [38,42,53,59] but autopsy consistently ruled out obstructive coronary disease as the primary cause of death. This supports prior observations that influenza-associated myocarditis often affects otherwise healthy individuals and that coronary artery disease is rarely a determinant of mortality in these presentations.

Influenza A was the predominant subtype, accounting for most adult and pediatric cases, while Influenza B was isolated in several severe and fatal myocarditis presentations [31,34,46,47]. This is aligned with prior epidemiological data indicating that both influenza A and B can precipitate fulminant myocarditis, though influenza A remains more frequently implicated in adults.

### 3.10. Influenza Vaccination Status

Across the 33 included case reports, the documentation of prior influenza vaccination was limited. A total of 12 cases clearly reported that the patient had not received the seasonal influenza vaccine during the relevant period. The remaining cases (*n* = 21) did not specify vaccination status. Notably, none of the reports indicated prior influenza vaccination, and no case described myocarditis occurring despite confirmed immunization.

The predominance of unvaccinated individuals among reports with available data suggests that influenza vaccination may have been a contributing factor in the susceptibility or severity of influenza-associated myocarditis. Additionally, comparing survival between Influenza A and B is challenging due to the low number of influenza B cases, making interpretation exploratory. Also, the high rate of missing vaccination data prevents strong conclusions about causality or risk.

## 4. Discussion

This systematic review synthesizes evidence from 33 published reports (32 case reports and one case series) describing influenza-associated cardiac manifestations. The findings are inherently influenced by publication bias, with a predominance of severe and fulminant cases, while mild or subclinical myocarditis is likely underrepresented. As the evidence is derived exclusively from case reports and case series, it does not permit the estimation of incidence, relative risk, or the proportion of influenza infections associated with cardiac complications. Importantly, the absence of mild cases in this review should not be interpreted as rarity but rather as a limitation of case-based reporting.

Despite these limitations, the included studies provide valuable insights into the clinical spectrum, underlying mechanisms, and outcomes of influenza-associated cardiac involvement. Reported manifestations ranged from mild myocardial inflammation to fulminant myocarditis, malignant arrhythmias, and sudden cardiac death. Notably, many cases occurred in previously healthy individuals without known coronary artery disease, supporting the possibility that influenza infection itself may contribute to acute cardiac complications [64].

This review indicates that influenza infection may be accompanied by acute cardiac events, most commonly myocarditis, including fulminant presentations with rapid hemodynamic deterioration [38]. Myocardial inflammation was frequently documented by histopathology, imaging, or clinical assessment, typically following a brief influenza-like prodrome [32,33,45,46,59]. Such manifestations were reported in both influenza A and B infections and across all age groups, including individuals without prior cardiovascular disease. Although definitive mechanisms cannot be established from case-based evidence, the close temporal association, inflammatory myocardial findings, and frequent requirement for intensive supportive care suggest acute viral or immune-mediated myocardial injury.

In some cases, cardiac events occurred in individuals with known or suspected cardiovascular disease, in whom influenza infection may have acted as a physiological stressor precipitating clinical decompensation [41,62]. These cases included patients with prior myocardial infarction, hypertension, arrhythmias, or heart failure, where influenza-related hypoxia, systemic inflammation, increased metabolic demand, or arrhythmogenic effects may have contributed to worsening cardiac function. Ischemic-appearing presentations, including ST-segment elevation and angiographic coronary abnormalities, were reported in several cases [24,32,37,50], raising the possibility of the unmasking or exacerbation of underlying coronary disease rather than isolated primary myocardial injury. However, inconsistent documentation of baseline cardiovascular status and limited coronary evaluation restrict definitive causal attribution.

Most patients initially presented with typical influenza-like symptoms, including fever, cough, and myalgia. However, rapid progression to myocarditis within hours to days of symptom onset was frequently observed, consistent with previous reports describing influenza-associated myocarditis as a highly variable and often unpredictable condition [3,63]. Atypical manifestations, such as gastrointestinal symptoms [64,65] or altered consciousness [66,67], were also reported and may obscure early recognition, particularly in children and young adults.

Adults aged 18–59 years accounted for more than three-fourths of reported cases, though fatalities occurred across all age groups, including young children. Influenza A, particularly the A/H1N1pdm2009 strain, was the predominant viral subtype implicated, consistent with previous literature linking this strain to severe systemic complications [68]. Influenza B–associated myocarditis was less frequently reported and appeared to be associated with comparatively better survival. The predominance of case reports from high-income countries likely reflects disparities in diagnostic capacity and surveillance rather than true epidemiologic differences.

Vital sign abnormalities, including tachycardia and tachypnea, were commonly documented and align with systemic inflammatory responses described in prior studies [69,70]. Notably, fever was absent in a substantial proportion of cases, challenging its reliability as a marker of disease severity.

Electrocardiographic findings were highly heterogeneous. While sinus tachycardia and ST-segment abnormalities were frequently observed [71,72], several patients demonstrated normal or non-specific ECGs, reinforcing that myocarditis cannot be excluded based on ECG findings alone [72]. Severe arrhythmias including ventricular tachycardia, ventricular fibrillation, and electromechanical dissociation were predominantly observed in fulminant cases and were often associated with cardiac arrest or mortality, consistent with prior reports of influenza-related fulminant myocarditis [1]. Brugada-like patterns were also reported, suggesting that influenza infection may unmask latent channelopathies or alter myocardial sodium-channel dynamics [72]. Autopsy findings demonstrated substantial variability, reflecting diverse mechanisms of cardiac injury. Acute lymphocytic myocarditis with myocyte necrosis and interstitial oedema was the most frequent finding among fatal cases, supporting immune-mediated or direct viral injury pathways. Similar observations have been reported in prior systematic reviews [3], with which our findings align, reaffirming myocarditis is the most common heart-related complication associated with influenza infection. This review dives deeper beyond previous work by offering a nuanced description of fulminant myocarditis, which accounted for 25% of cases, and sudden cardiac death, observed in 17% of cases. These severe outcomes were not highlighted to the same extent in earlier studies. Importantly, our analysis includes a notable proportion of influenza B cases, revealing that the risk of serious cardiac complications extends beyond influenza A. This broader perspective clarifies distinctions that were less apparent in prior reviews.

In other fatal cases, severe pulmonary pathology including hemorrhagic bronchiolitis, diffuse alveolar damage, or bacterial superinfection appeared to be the dominant cause of death, highlighting the complex interplay between respiratory and cardiac failure. Ischemic lesions were less common and may reflect influenza-induced prothrombotic states or coincidental coronary pathology.

A notable observation was that outcomes did not correlate with pre-existing cardiac disease. Several deaths occurred in individuals with structurally normal hearts, underscoring that influenza-associated myocarditis is primarily driven by acute inflammatory injury rather than chronic coronary disease.

Vaccination status was poorly documented; however, among cases with available data, most patients were unvaccinated, and none had confirmed prior influenza vaccination. Although conclusions are limited by missing data, this pattern aligns with population-level evidence demonstrating reduced cardiovascular complications among vaccinated individuals [56].

The findings of this systematic review should be interpreted in the context of the risk of bias of the included studies, which were predominantly case reports with one case series. Using the Joanna Briggs Institute (JBI) critical appraisal tools, most studies demonstrated adequate reporting of clinical presentation and diagnostic confirmation; however, incomplete reporting across selected domains, particularly those related to therapeutic interventions, post-intervention outcomes, follow-up duration, and adverse events, was observed in several reports. These limitations resulted in some concerns for risk of bias, driven largely by reporting deficiencies rather than clear methodological flaws, a common limitation of case-based evidence. The single included case series demonstrated generally acceptable methodological quality, with only one appraisal item not applicable, further supporting the descriptive strength of the evidence. Consequently, while the review provides valuable insights into rare clinical manifestations, the overall certainty of evidence remains limited, and the findings should be regarded as hypothesis-generating, highlighting the need for prospective, systematically reported studies.

Overall, the findings of this study underscore the importance of the early recognition of myocarditis in influenza patients presenting with chest pain, dyspnea, unexplained tachycardia, syncope, or hemodynamic instability. ECG and troponin testing should be considered routine but not definitive, and echocardiography and cardiac MRI remain essential diagnostic tools. In fulminant cases, the early initiation of advanced supportive therapies, including mechanical circulatory support, may be life-saving. Despite its rarity, influenza-associated myocarditis remains one of the most dramatic and life-threatening complications of influenza infection.

### Limitations

This review provides a detailed synthesis of published case-level evidence on influenza-associated myocarditis; however, several limitations must be considered. Case reports are inherently prone to publication bias, with a tendency to emphasize severe, atypical, or fulminant presentations, while milder cases remain underreported. Incomplete documentation, particularly regarding vital signs, diagnostic investigations, treatment timelines, and vaccination history, further restricts the ability to perform consistent quantitative assessments. Considerable heterogeneity in diagnostic criteria, ranging from clinical suspicion alone to biopsy- or MRI-confirmed myocarditis, complicates direct comparison across cases and limits generalizability. Moreover, the absence of controlled studies prevents the accurate estimation of causal risk factors, disease severity distribution, or true incidence in the broader population. Despite these constraints, case-level evidence continues to offer critical insights into rare but clinically significant manifestations of influenza infection and highlights patterns that may inform early recognition and management. Also, the inclusion of papers published only in English language may introduce selection bias.

Additionally, survival, publication, and language biases likely resulted in the overrepresentation of severe and fatal cases. The findings of this review should also be interpreted in light of the inherently low certainty of evidence associated with case reports and case series. These study designs are highly susceptible to publication bias, selective reporting, incomplete clinical documentation, and a lack of comparators, limiting causal inference and generalizability. Consequently, the observations synthesized in this review should be regarded as hypothesis-generating and descriptive rather than definitive evidence of incidence or risk.

Also, this review did not systematically analyze pharmacological management strategies, including antiviral therapy, supportive treatment, or cardiac-specific interventions, as the primary focus was the characterization of influenza-associated cardiac manifestations and outcomes. Therapeutic approaches were heterogeneous and inconsistently reported across case reports, limiting meaningful comparative analysis. Future studies focusing on treatment patterns and their impact on clinical outcomes would be valuable.

These limitations highlight the need for prospective, population-based studies with standardized diagnostic criteria to better define the cardiovascular risk associated with influenza infection.

## 5. Conclusions

This systematic review brings together published case-based evidence describing acute cardiac manifestations. Across the included reports, influenza A was associated with a broad spectrum of cardiac manifestations, ranging from acute and fulminant myocarditis to cardiogenic shock, heart failure, malignant arrhythmias, ST-segment elevation, acute myocardial infarction, and sudden cardiac death. Influenza B demonstrated a largely overlapping clinical profile, with reported presentations including myocarditis (including fulminant forms), myopericarditis, cardiogenic shock, heart failure, ventricular arrhythmias, cardiac arrest, and fatal myocardial involvement. Although influenza A cases were more frequently reported, influenza B was associated with a comparable severity spectrum, suggesting no clear qualitative difference in cardiac presentations between the two virus types based on the available data.

While some cases occurred in individuals without known cardiovascular disease and others in those with underlying or subclinical cardiac conditions, the available evidence does not permit a definitive attribution of causality or estimation of risk magnitude. Substantial heterogeneity in clinical presentations, diagnostic methods, and reporting standards further limits robust conclusions regarding underlying mechanisms or subtype-specific effects. Nevertheless, the recurrent reporting of myocardial inflammation, arrhythmias, hemodynamic instability, and cardiac failure across diverse clinical settings suggests that cardiac involvement should be recognized as part of the broader clinical spectrum of influenza illness. Given the predominance of case reports and case series, well-designed prospective, population-based studies incorporating standardized diagnostic criteria and comprehensive cardiovascular assessment are needed to better define the nature, frequency, and clinical relevance of influenza-associated cardiac manifestations and to inform evidence-based prevention and management strategies.

From a public health perspective, these findings underscore the importance of improved documentation of influenza vaccination status, standardized reporting of cardiac outcomes, and more systematic evaluation of cardiac involvement during influenza infection.

## Figures and Tables

**Table 2:** Cardiac manifestations reported in included studies.

Cardiac Event Category	Description	Percent (%)	References
Fulminant myocarditis	Rapid-onset severe myocarditis with hemodynamic collapse, often requiring vasopressors/ECMO	9 (25%)	Shakerian et al., 2024; Taremi et al., 2013; Frank et al., 2010; Baik et al., 2015; Lobo et al. 2014; Montcriol et al. 2008; Cabral et al. 2012; Kovačević et al. 2019; Ona et al. 2012 [19,31,40,51,55,57,58,61]
Non-fulminant myocarditis/Myocardial inflammation	Biopsy/MRI-confirmed myocarditis without fulminant deterioration	12 (33%)	Erden et al. 2011; Marchetti et al. 2019; Chang et al. 2016; Siskin et al. 2017; Roto et al. 2018; Dickey et al. 2018; Wattanachayakul et al. 2024; Muneuchi et al. 2009; Murnaghan et al. 2022; Mazzitelli et al. 2019; Chang et al. 2020; Gdynia et al. 2011 [32,34,35,44,45,46,47,50,52,54,56,59]
Myocardial infarction/STEMI-like presentation	ST-elevation, troponin rise, angiographic stenosis, or normal coronaries	3 (8%)	Iwanaga et al. 2014; Ishiguro et al. 2019; Arbit et al. 2017 [24,37,52]
Arrhythmias	Sustained VT, VF, tachyarrhythmia	4 (11%)	Dickey et al., 2018; Wiegand et al. 2011b, Wiegand et al. 2011c, Wigand et al. 2011a [38,47]
Cardiogenic shock	Shock requiring vasopressors or mechanical support	7 (19%)	Shakerian and Mandegar 2024; Marchetti et al. 2019); Siskin et al. 2017; Taremi et al. 2013; Murnaghan et al. 2022; Wiegand et al. 2011a, Wiegand et al. 2011b [34,38,40,45,47,51,54]
Cardiac arrest/sudden cardiac death	Asystole, pulseless electrical activity, sudden death	6 (17%)	Okada et al. 2017; Ono et al. 2023; Callon et al. 2021; Boulagnon et al. 2012; Ebdrup et al. 2018; Tian et al. 2024 [33,36,42,43,49,53]

**Table 3:** Summary of electrocardiographic abnormalities reported in case studies of influenza-associated myocarditis.

ECG Pattern	Description	Number of Cases (%)	References
ST-segment elevation (any territory)	Localized or diffuse STE across inferior, anterior, lateral, or mixed leads	14 (38.89%)	Iwanaga et al. 2014; Shakerian et al., 2024; Erden et al., 2010; Ishiguroa et al., 2019; Ono et al., 2023; Siskin et al., 2017; Dickey et al., 2018; Wattanachayakul et al., 2024; Tian et al., 2024; Cabral et al., 2012, Kovačević et al., 2019; Arbit et al., 2017; Gdynia et al., 2011; Mel A. Ona, et al. 2012 [24,32,37,40,41,43,45,47,50,53,58,59,61]
Sinus tachycardia	With or without ST-T changes	9 (25%)	Shakerian et al., 2024; Chang et al. 2016; Siskin et al., 2017; Roto et al., 2018; Wattanachayakul et al., 2024; Murnaghan et al., 2022; Lobo et al., 2014; Kovačević et al., 2019; Mel A. Ona, et al. 2012; [40,43,44,45,48,51,56,62]
Reciprocal ST-segment depression	Typically accompanying inferior STE	3 (8.33%)	Shakerian et al., 2024; Erden et al., 2010; Muneuchi et al., 2009 [32,40,52]
Wide-complex tachycardia/ventricular tachyarrhythmia	Including ventricular tachycardia	2 (5.56%)	Iwanaga et al. 2014; Mel A. Ona, et al. 2012 [24,61]
Brugada-pattern ECG	Type 1 Brugada-like pattern	1 (2.78%)	Ono et al., 2023 [43]
Right bundle branch block (RBBB)	With or without STE (V1–V2)	2 (5.56%)	Ono et al., 2023; Cabral et al., 2012 [43,58]
Low QRS voltage	Reduced amplitude in precordial leads	2 (5.56%)	Baik et al., 2015 [19]
Frequent PVCs	Documented on ECG	1 (2.78%)	Murnaghan et al., 2022 [54]
Electromechanical dissociation (EMD)	Diffuse broad complexes with pulseless collapse	1 (2.78%)	Ishiguroa et al., 2019 [41]
Normal ECG	No ST changes, no arrhythmias	3 (8.33%)	Mazzitelli et al., 2019; Chang et al., 2020; Montcriol et al., 2008 [35,56,57]
ECG Not Reported/Not Available	Mentioned but not described	6 (16.67%)	Okada et al., 2017; Callon et al., 2021; Boulagnon et al., 2012; Ebdrup et al., 2018; Wiegand et al., 2011; Rivera-Guzmán et al., 2016 [33,36,38,42,49,60]

## Data Availability

The data that support the findings of this study are available from the corresponding author upon reasonable request.

## Supplementary Materials

The following supporting information can be downloaded at: https://www.mdpi.com/article/10.3390/jcm15166243/s1, Table S1: Frequency of clinical symptoms among reported cases of influenza-associated cardiac events; Table S2: Vital signs of the cases included; Table S3: PRISMA Checklist.
