# Supplementary material for: Influenza-Associated Cardiac Manifestations: A Systematic Review of Case Reports and Case Series
Source: J Clin Med. 2026 Aug 12;15(16):6243. doi: 10.3390/jcm15166243 (PMC13513689; doi:10.3390/jcm15166243)
Supplement: Supplementary file 1 [file jcm-15-06243-s001.zip › jcm-4306755 - SM Tables.pdf]

**Table S1.** Frequency of clinical symptoms among reported cases of influenza-associated cardiac events.

| S. No | Author (Year) | Fever | Cough | Sore Throat / URTI | Myalgia / Malaise | Dyspnea | Chest Pain | GI Symptoms | Neuro / Consciousness | Other Notes |
|---|---|---|---|---|---|---|---|---|---|---|
| 1 | Iwanaga et al 2014 | Yes | NR | NR | Flu-like | NR | NR | NR | NR | Flu-like symptoms |
| 2 | Shakerian et al, 2024 | Yes | NR | NR | Fatigue + myalgia | Yes | NR | NR | NR | Flu-like symptoms |
| 3 | Erden et al, 2010 | Yes | Yes | Sore throat | Myalgia | Yes | NR | Nausea | NR | – |
| 4 | Ishiguroa et al, 2019 | Yes | Yes | NR | NR | Yes | NR | NR | NR | – |
| 5 | Okada et al, 2017 | Yes | NR | NR | NR | Yes | NR | NR | NR | – |
| 6 | Ono et al, 2023 | Yes | NR | NR | Malaise | NR | NR | NR | NR | – |
| 7 | Callon et al, 2021 | Yes | NR | NR | Flu-like | NR | NR | NR | NR | Flu-like syndrome |
| 8 | Boulagnon et al, 2012 | Yes | NR | NR | NR | NR | NR | NR | NR | – |
| 9 | Marchetti et al, 2019 | Yes | Yes | NR | Myalgia + fatigue | NR | NR | NR | NR | – |
| 10 | Chang et al. 2016 | High fever | NR | Throat pain | Myalgia | NR | NR | Poor appetite | NR | Chills |
| 11 | Siskin et al, 2017 | Yes | URTI | URTI | NR | NR | NR | NR | NR | Chills |
| 12 | Roto et al, 2018 | Fevers (intermittent) | Mild cough | NR | NR | NR | NR | NR | NR | UTI |
| 13 | Dickey et al, 2018 | Low-grade fever | Cough | NR | Malaise | NR | Epigastric pain | Yes (epigastric) | NR | fever self reported |
| 14 | Hollowed et al, 2019 | NR | NR | NR | NR | NR | NR | Abdominal pain, N/V/D | No | GI predominant |
| 15 | Ebdrup et al, 2018 | NR | NR | NR | NR | NR | NR | Abdominal pain | NR | Capillary leak syndrome |
| 16 | Wattanachayakul et al, 2024 | Afebrile | Productive cough | NR | NR | Yes | Yes | NR | NR | Chest pain |
| 17 | Taremi et al, 2013 | Subjective fever | Productive cough | NR | Myalgia | NR | NR | NR | NR | Chills |
| 18 | Muneuchi et al, 2009 | NR | NR | NR | NR | NR | Yes | NR | NR | Acute chest pain |

| | | | | | | | | | | |
|---|---|---|---|---|---|---|---|---|---|---|
| 19 | Tian et al, 2024 | Intermittent fever | NR | NR | NR | NR | NR | NR | NR | – |
| 20 | Frank et al, 2010 | Yes (39°C) | Cough | URTI | NR | NR | NR | NR | NR | URTI + rhinitis |
| 21 | Murnaghan et al, 2022 | Yes | Productive cough | NR | Myalgia | NR | NR | NR | NR | Chills |
| 22 | Baik et al, 2015 | NR | Severe productive cough | NR | Myalgia | NR | NR | NR | NR | Hemoptysis |
| 23 | Mazzitelli et al, 2019 | Yes | Yes | NR | NR | Yes | NR | NR | NR | Shortness of breath |
| 24 | Lobo et al, 2014 | Yes | Cough | Rhinorrhea | Poor condition | NR | NR | Abdominal pain + vomiting | NR | --- |
| 25 | Chang et al, 2020 | Yes | Yes | Sore throat + rhinorrhea | NR | NR | NR | NR | NR | Pregnant woman |
| 26 | Montcriol et al, 2008 | NR | NR | NR | Flu-like | No | NR | NR | Altered consciousness | Influenza like |
| 27 | Cabral et al, 2012 | Yes | Yes | NR | Myalgia | NR | NR | Vomiting + diarrhea | Headache | |
| 28a | Wiegand et al, 2011 | Yes | NR | NR | Myalgia | Yes | NR | NR | Headache | – |
| 28b | Wiegand et al, 2011 | Yes | Yes | NR | Fatigue | NR | NR | NR | Headache | – |
| 28c | Wiegand et al, 2011 | Flu-like | NR | NR | NR | NR | NR | NR | NR | – |
| 28d | Wiegand et al, 2011 | Yes | Yes | URTI | NR | NR | NR | NR | NR | Chills |
| 29 | Mila Kovačević et al , 2019 | No measured fever | NR | NR | Flu-like | NR | NR | Excessive sweating | NR | – |
| 30 | Boris ARBIT et al , 2017 | Yes | NR | Sore throat | NR | Yes | Yes | Nausea + diarrhea | NR | Acute MI |
| 31 | Georg Gdynia et al ,2011 | Mild fever | Minor cough | Rhinitis + pharyngitis | Malaise | NR | NR | Diarrhea | NR | URTI |
| 32 | Norwin Rivera-Guzmán et al , 2016 | Unquantified fever | Yes | NR | Arthralgia | Yes | Chest discomfort | NR | NR | – |
| 33 | Mel A. Ona, et al 2012 | NR | Yes | Sore throat + rhinorrhea | Malaise | NR | NR | Nausea | NR | Chills |

**Table S2.** Vital signs of the cases included.

| S. No. | Authors | HR | BP | Temp | RR | $SpO_2$ |
|---|---|---|---|---|---|---|
| **1** | Iwanaga et al 2014 | 120 | 122/81 | 40 | 32 | NR |
| **2** | Shakerian et al, 2024 | 120 | 70/50 | 35.9 | 28 | 92 |
| **3** | Erden et al, 2010 | 92 | 112/72 | 37.6 | 24 | 94 |
| **4** | Ishiguroa et al, 2019 | 129 | 115/66 | 37.7 | 38 | NR |
| **5** | Okada et al, 2017 | 129 | 115/66 | 37.7 | 38 | NR |
| **6** | Ono et al, 2023 | NR | 115/66 | NR | 38 | NR |
| **7** | Callon et al, 2021 | NR | NR | NR | NR | NR |
| **8** | Boulagnon et al, 2012 | NR | NR | 38.4 | NR | NR |
| **9** | Marchetti et al, 2019 | NR | NR | NR | NR | NR |
| **10** | Chang et al. 2016 | 112 | 120/71 | 40 | NR | NR |
| **11** | Siskin et al, 2017 | 120 | 90/75 | NR | 20 | 100 |
| **12** | Roto et al, 2018 | 120 | 58/41 | 32.4 | NR | NR |
| **13** | Dickey et al, 2018 | 71 | 136/93 | NR | 20 | 98 |
| **14** | Hollowed et al, 2019 | NR | 30/NR | NR | NR | NR |
| **15** | Ebdrup et al, 2018 | NR | NR | NR | NR | NR |
| **16** | Wattanachayakul et al, 2024 | NR | NR | NR | NR | NR |
| **17** | Taremi et al, 2013 | 115 | 90/60 | NR | NR | NR |
| **18** | Muneuchi et al, 2009 | 80 | 120/65 | 37.2 | NR | NR |
| **19** | Tian et al, 2024 | 168 | NR | 36.4 | NR | NR |
| **20** | Frank et al, 2010 | NR | NR | 36.4 | NR | 96 |
| **21** | Murnaghan et al, 2022 | 130 | 131/98 | 37.1 | 22 | 99 |
| **22** | Baik et al, 2015 | 114 | 91/59 | 37.9 | 32 | NR |
| **23** | Mazzitelli et al, 2019 | NR | NR | NR | NR | NR |
| **24** | Lobo et al, 2014 | 166 | NR | NR | NR | NR |
| **25** | Chang et al, 2020 | 100 | 110/70 | 37.9 | 20 | 100 |
| **26** | Montcriol et al, 2008 | 120 | 70/30 | NR | NR | NR |
| **27** | Cabral et al, 2012 | NR | NR | 39.3 | NR | NR |
| **28** | Wiegand et al, 2011 | NR | 75/58 | NR | NR | NR |
| | Wiegand et al, 2011 | NR | 62/45 | NR | NR | NR |
| | Wiegand et al, 2011 | NR | 100/70 | NR | NR | NR |
| | Wiegand et al, 2011 | NR | NR | NR | NR | NR |
| **29** | Mila Kovačević et al , 2019 | 120 | 120/80 | 35.9 | 30 | 92 |
| **30** | Boris ARBIT et al , 2017 | 58 | 115/72 | NR | 18 | 97 |
| **31** | Georg Gdynia et al ,2011 | 80 | 110/70 | NR | NR | NR |
| **32** | Norwin Rivera-Guzmán et al , 2016 | 50 | 146/61 | NR | 18 | NR |
| **33** | Mel A. Ona, et al 2012 | 120 | 79/56 | NR | NR | NR |
